# Novel anti-arthritic mechanisms of trans-cinnamaldehyde against complete Freund’s adjuvant-induced arthritis in mice: involvement of NF-кB/TNF-α and IL-6/IL-23/ IL-17 pathways in the immuno-inflammatory responses

**DOI:** 10.1007/s10787-022-01005-y

**Published:** 2022-06-01

**Authors:** Ghada S. El-Tanbouly, Rehab S. Abdelrahman

**Affiliations:** 1 Department of Pharmacology, Faculty of Pharmacy, Delta University for Science and Technology, Gamasa, 11152 Egypt; 2 Department of Pharmacology and Toxicology, Faculty of Pharmacy, Mansoura University, Mansoura, 35516 Egypt; 3 Department of Pharmacology and Toxicology, College of Pharmacy, Taibah University, Al-Madina Al-Munawwarah, 30001 Saudi Arabia

**Keywords:** Cinnamaldehyde, Adjuvant, Arthritis, NF-κB, IL-17, IL-23

## Abstract

Trans-cinnamaldehyde (TCA), a natural cinnamaldehyde derivative of cinnamon oil, is known for anti-inflammatory, anti-bacterial, anti-fungal, anti-diabetic, and anti-cancer activities. However, no study has examined the protective mechanisms of TCA on complete Freund’s adjuvant (CFA)-induced arthritis. Chronic arthritis was induced in mice by triple dose injection of 0.1 ml CFA in the first two days, then a treatment with TCA (100 mg/kg, i.p.) and the anti-arthritic drug; methotrexate (MTX, 0.75 mg/kg, i.p., 3 times/week) started from day 10 after CFA and continued till day 35.TCA ameliorated the CFA-induced arthritis features, indicated by the decrease in serum rheumatoid factor, paw swelling, arthritis index and the arthritis changes in limb histology. Additionally, TCA treatment showed anti-inflammatory actions through downregulation of TNF-α, NF-κB and COX-2 expressions and marked reduction in IL-1β, IL-6, IL-23 and IL-17 levels in inflamed paw tissues.Consequently, TCA can decrease arthritis progression and inhibit the immune/inflammatory responses initiated by TNF-α/IL-1β/IL-6/IL-23/IL-17 signals, via NF-κB modulation, almost to the same extent accomplished by MTX. Therefore, TCA could be a promising anti-arthritic drug.

## Introduction

Rheumatoid arthritis (RA) is an autoimmune disease, characterized by chronic and symmetric inflammation of synovial joints that leads to stages of pathological process (Xu et al. 2017). The early stage shows symptoms of heat, painful swelling, and decreased joint function; the late-stage shows various degrees of joint stiffness and deformity accompanying chronic joint pain, loss of function, joint destruction, bone damage, and disability risk (Huffman et al. 2017). The pathogenesis and precise mechanisms by which RA is initiated, and aggravated, are poorly understood. Previously, a partial explanation of the RA pathogenesis was documented as a detention of microbial products in the synovial membrane and continual infection of the joint articulation, which initiates an immune reaction, eventually leading to modification of the structural integrity of the joint (Walsh and McWilliams 2014). For this reason, complete Freund’s adjuvant (CFA), heat-killed mycobacterium tuberculosis, has been successfully used in animals to generate inflammation in the affected joints, resembling the arthritis profile in humans (Robledo-González et al. 2017).

The aggressive and complex involvement of immune cells, pro-inflammatory cytokines, and other inflammatory mediators in RA pathogenesis is confirmed (Sokolove et al. 2014). Various inflammatory cells infiltrate the affected inflammatory sites, releasing the pro-inflammatory cytokines; tumor necrosis factor-alpha (TNF-α), interleukin-1β (IL-1β), interleukin-6 (IL-6), and interleukin-17 (IL-17). TNF-α and IL-1β could amplify the inflammatory responses in RA (Fouser et al. 2008). IL-6 could enhance the differentiation and maturation of T helper-17 lymphocytes (Th-17) to produce IL-17, IL-6, and TNF-α (Kamel et al. 2018). In addition, IL-23, a central cytokine in autoimmunity, is also present in RA inflammatory sites, and is required for Th-17 proliferation. Thereby, enhances Th-17 cells to release IL-17, in addition to activating the release of IL-1, TNF-α, and IL-23 itself (Duvallet et al. 2011). IL-17 plays a pivotal role in the progression of RA and contributes to edema, swelling, oxidative stress, and inflammation. IL-17 increases reactive oxygen species (ROS) production, and activates nuclear factor (NF-κB) expression and its related downstream cascade, including the inflammatory mediators: tumor necrosis factor-alpha (TNF-α), and interleukin (IL-1β), in addition to cycloxygenase (COX-2) enzyme (Kamel et al. 2018).

The complication of immune/inflammatory interplay could open the way to the development of new therapy for RA on an effective mechanistic basis. Currently, there is no effective cure for RA. Most common therapies include nonsteroidal anti-inflammatory drugs, glucocorticosteroids, anti-TNF, anti-CD20 therapy, and CD80/86 blockade. However, chronic administration of these agents is associated with limited effectiveness and numerous side effects (Schiff et al. 2011; Harmse and Reuter 2016). In light of this, there is an urgent clinical need to promote mechanism-based therapies with fewer side effects and more effective targets for analgesic and anti-inflammatory effects to treat joint pain in patients with RA. Therefore, exploration of traditional herbal drugs that are more efficient, safer, and economic, has attracted serious attention, as, 80% of the universal population, primarily consume herbal drugs in their food (Murunikkara and Rasool 2017).

Cinnamaldehyde (CA) is the main bioactive ingredient of cinnamon oils derived from the bark extract of cinnamon and is responsible for the odor and flavor of cinnamon. Therefore, it is processed as a natural fragrant and flavoring agent in the kitchen and industry (Zhu et al. 2017). Natural CA, and its derivatives, including trans-cinnamaldehyde (TCA), have numerous biological activities, involving anti-bacterial, anti-fungal, anti-cancer, anti-diabetic, anti-platelet aggregation, peripheral vasodilatation, anti-inflammatory, anti-apoptotic, anti-mutagenic and neuroprotective properties (Huang et al. 2007; Zhu et al. 2017, Deng et al. 2018). To this date,there is no study of the effect of TCA on CFA-induced arthritis in mice.

Therefore, CFA-induced arthritis is performed on mice to delineate the immune/inflammatory pathomechanisms in arthritis-associated severity, and to evaluate the potential counter-regulatory function, and activation mechanisms exerted by TCA, the main natural CA derivative, in comparison with a known anti-arthritic drug; methotrexate (MTX).

## Materials and methods

### Chemicals

The following compounds are used in the study, and are obtained from the sources indicated:

CFA (1 mg heat-killed Mycobacterium tuberculosis/ml paraffin oil) and Cinnamaldehyde (trans-cinnamaldehyde (TCA), liquid form, re-dissolved in 0.5% (w/v) carboxymethyl cellulose (CMC) in water), are obtained from (Sigma-Aldrich, St. Louis, MO, USA). Methotrexate (MTX; Unitrexate, 25 mg/ml) is obtained from (Hikma Specialized pharmaceuticals, Egypt) and diluted with normal saline. All other chemicals are of the finest analytical grade.

### Animals

A total of thirty BALB/c male mice, weighing 25–30 g (10 weeks old) is used. Animals were kept under standard conditions throughout the experiment, and were allowed free access to food and water.

### Complete Freund’s adjuvant (CFA)-induced arthritis

Chronic arthritis was induced by a single intraplantar injection of 0.1 ml of CFA into the right hind limb, and one hour later, another subcutaneous (s.c.) injection of 0.1 mL CFA was implemented into the tail root. On the following day, another s.c. booster dose of 0.1 mL CFA was injected into the tail root to potentiate the systemic effects (Kamel et al. 2018). The development of arthritis is monitored weekly by measuring the paw thickness throughout the thiry five days experimental period. Meanwhile, an equivalent volume of normal saline was injected into the mice in the normal control group (Xu et al. 2017). Ten days after CFA injection, drugs were tested, as inflammation, and paw edema reach a maximum point at this time period. On day 35, mice were killed by euthanasia.

### Experimental design

Mice were randomly divided into five groups (*n* = 6/each) (1) Control group: received paraffin oil in the right hind limb, equivalent to the volume injected with CFA, then received 0.5% (w/v) CMC in water, i.p., as a vehicle for TCA, from day 10 till the end of the study. (2) Trans-cinnamaldehyde (TCA) control group: received TCA (100 mg/kg, in 0.5% (w/v) CMC in water, i.p.) from day 10 till the end of the study. (3) Complete adjuvant-induced arthritis (CFA) group: received 0.3 ml CFA, as indicated before. (4) Methotrexate (CFA/MTX) group: received 0.3 ml CFA as indicated before, then MTX (0.75 mg/kg, i.p., 3 times in a week) from day 10 till the end of the study. (5) Complete adjuvant-induced arthritis + Trans-cinnamaldehyde (CFA/TCA) group: received 0.3 ml CFA as indicated before, then TCA (100 mg/kg, in 0.5% (w/v) CMC in water, i.p.) from day 10 till the end of the study.

Doses of TCA and MTX were chosen based on previous studies (Huang et al. 2007) and (Kamel et al. 2018), respectively. Mice were anaesthetized with thiopental and blood was obtained from a retro-orbital puncture, 24 h after the last TCA administration, then centrifugated to collect the sera for further biochemical estimations. The right hind paw was immediately dissected and divided into two parts. One part was homogenized using (10% w/v) phosphate-buffered saline (50 mM K_2_HPO_4_, pH 7.5) for the assay of ELISA experiments. The other part was fixed in 10% buffered formalin for histopathological and immunohistochemical analyses.

### Evaluation of CFA-induced macroscopical arthritis changes (arthritis index, and paw thickness)

The severity of arthritis was assessed visually by an independent observer. Mice were observed weekly for the severity of joint inflammation for a total of 5 weeks. The severity of arthritis was graded on modified five-point scale (Hegen et al. 2008); *4:* indicating severe edema and inflammation with hardness in movement, 3: indicating moderate to severe edema and erythema, *2:* indicating mild edema and erythema, *1:* indicating no edema and limited erythema, and *0:* indicating no edema or erythema. The arthritis index for each mouse is the sum of the severity in both hind paws (maximum index = 8 points) (Bendele 2001).

Measurements of the paw swelling was recorded on days 0, and day 1 after the CFA injection, then every 7 days till the end of the experiment (days 7, 14, 21, 28, and 35). The paw thickness diameter just below the level of the foot was measured in the anesthetized mice using a digital caliper vernier scale. The measurement was repeated three times and the average value was used. The paw swelling was calculated using the following equation: paw swelling degree = (paw thickness after CFA—paw thickness before CFA). The results are expressed as (mm^2^) (Xu et al. 2017).

### Assessment of rheumatoid factor (RF) in serum

The serum was used immediately for determination of the arthritis marker; RF (SPINREACT, Sant Esteve de Bas, Spain, catalog number: SGIS02-I), according to the manufacturer’s instructions.

### Histopathological assessment of arthritis scores of the hind limb

Formalin-fixed paw tissues were decalcified with a solution containing hydrochloric acid and 0.1 M EDTA, then tissues were embedded in paraffin, sectioned, and stained with hematoxylin–eosin (H&E) (Shen et al. 2013a). Histopathological assessments were double executed blindly by a pathologist.

The severity of arthritis was examined in right hind paw sections, using a semi-quantitative scoring system on a 4-point scale (0: absent, 1: mild, 2: moderate, 3: severe) (Snekhalatha et al. 2013). The following parameters were assessed: enlargement of synovial lining cell layer, synovial hyperplasia, synovial inflammation, synovial vascularity proliferation. Then pannus formation, bone and cartilage erosion were observed in the joint of the mouse (maximum score = 21/section). Finally, total histopathological scores were calculated for each group, and compared with controls.

### Immunohistochemistry for NF-κB p65, TNF-α, and COX-2 in hind limb

The expressions of NF-κB p65, TNF-α, and COX-2 were assessed in paw tissue sections immuno-stained with antibodies for NF-κB p65 (Bioss antibodies, MA, USA, catalog number: bs-20159R), TNF-α (ABclonal Technology, MA, USA, catalog number: A0277), and COX-2 (ABclonal Technology, MA, USA, catalog number: A1253). Avidin–Biotin Complex (ABC) method was used according to the manufacturer’s protocol. For scoring nuclear expression, semi-quantitative analysis was performed in two random fields per section, and compared with controls.

### ELISA measurements (IL-1β, IL-6, IL-23, and IL-17)

The paw homogenate was used for the estimation of interleukins concentrations (IL-1β, IL-6, IL-23, and IL-17) by sandwich enzyme-linked immunosorbent assay (sandwich ELISA) using commercial kits (Bioassay Technology Laboratory, Shanghai Crystal Day Biotech Co., Ltd., Shanghai, China), following the producer’s instructions. Briefly, plates were filled with a biotinylated antibody reagent, followed by the serum homogenate, then incubated at 37^∘^C for 2 h. Phosphate-buffered saline (PBS) was used for washing, then the plates were filled with streptavidinhorseradish peroxidase (HRP) solution, followed by another 30 min incubation period. Absorbance was set at 450 nm with a microplate reader.

#### Statistical analysis

Results were expressed as means ± S.E.M. Statistics and graphical representation were carried out using Graphpad Prism V 6.01 (Graphpad Software Inc., San Diego, CA, USA) by regular one-way ANOVA followed by Tukey's post hoc test for multiple comparisons. Repeated measures two-way ANOVA followed by Bonferroni post hoc multiple comparison test was used for analyzing arthritis index, and paw thickness. Kruskal–Wallis test followed by Dunn's multiple comparison test was used for RF, histopathological and immunohistochemical scoring. Statistical significance was set at *p* < 0.05.

## Results

### The impact of TCA on arthritis changes (arthritis index and paw thickness)

**Fig. 1:**
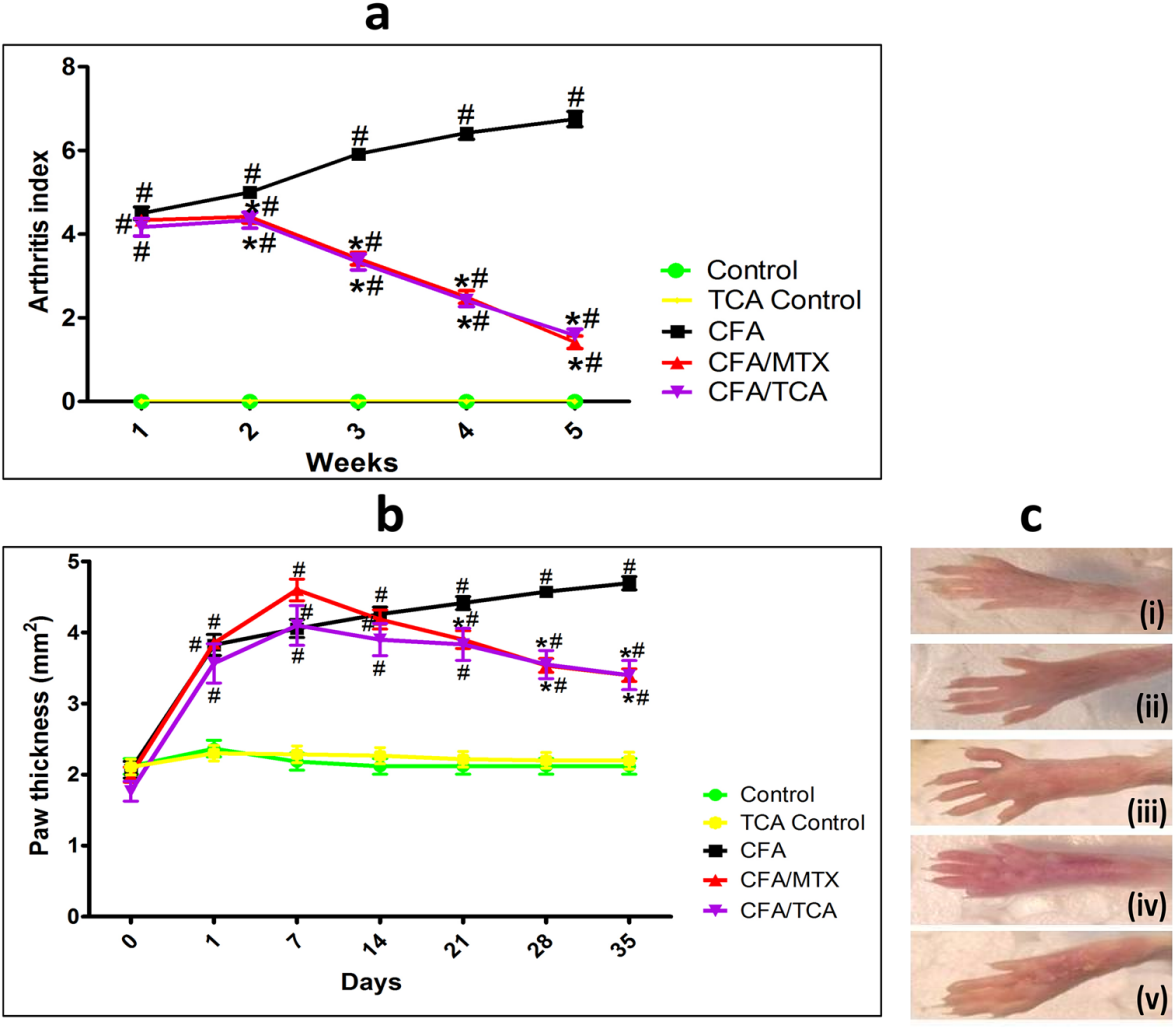
TCA improved the arthritis-induced morphological manifestations In CFA-induced arthritis model for 5 weeks, mice were periodically observed for the severity of joint inflammation and swelling. Arthritis index (**a**), and paw thickness (**b**) were measured in CFA-induced arthritis mice model. Data are represented as mean ± S.E.M. (*n* = 5). Statistical analysis was performed using Repeated measures two-way analysis of variance (ANOVA) test, followed by Bonferroni post hoc multiple comparison test, #, *p < 0.05 vs. control, CFA and MTX groups, respectively. Macroscopic images of hind limbs (**c**) are presented for (i) control, (ii) TCA control, (iii) CFA, (iv) CFA/MTX, and (v) CFA/TCA mice. *CFA* complete freund’s adjuvant, *MTX* methotrexate, *TCA* trans-cinnamaldehyde

TCA control group did not experience any arthritis changes (Fig. 1a–c). The observed RA morphological severity in the arthritis index is significant in CFA treated group. Both CFA/MTX and CFA/TCA treated groups, could effeciently decrease the morphological severity, to the same extent, reflected by a signifcant variation in the arthritis index, compared with the CFA group. (Fig. 1a).

The paw swelling in the CFA and treated groups is gradually and signifcantly elevated compared to the control group (*p* < 0.05) starting from the 1st week, indicating that RA is established correctlly. On the 4th and 5th weeks, the paw swelling was significantly reduced in CFA/MTX and CFA/TCA treated groups, to the same extent, compared with the CFA group (Fig. 1b, c).

### The impact of TCA on serum RF

**Fig. 2:**
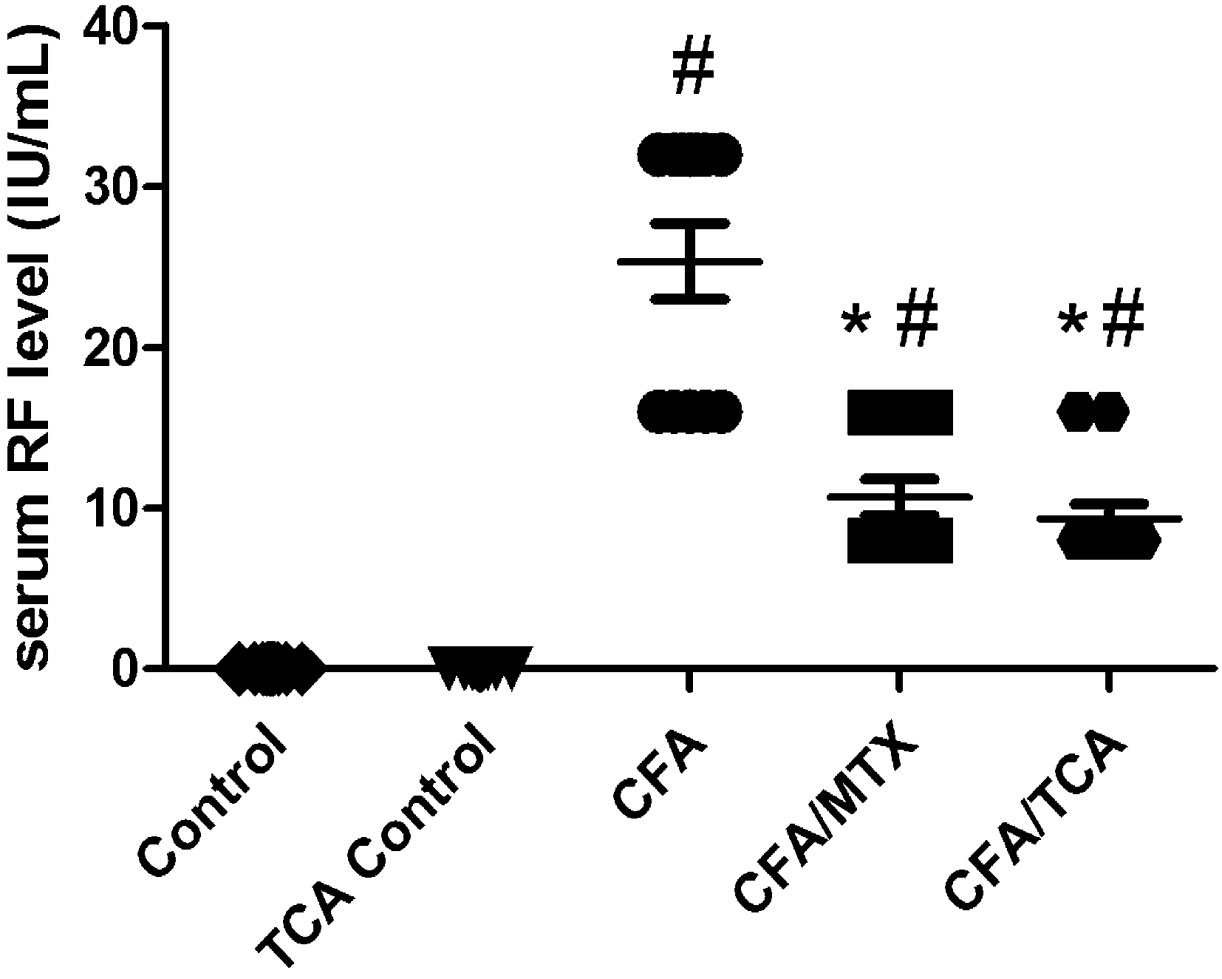
Effect of TCA on serum RF Serum levels of the arthritis marker: RF, was measured in CFA-induced arthritis mice model after 5 weeks. Data are presented as median. (*n* = 6). Statistical analysis was performed using Kruskal–Wallis test followed by Dunn's multiple comparison test, #, *p < 0.05 vs. control and CFA groups, respectively, *CFA* complete freund’s adjuvant, *MTX* methotrexate, *TCA* trans-cinnamaldehyde, *RF* rheumatoid factor

CFA produced a significant increase in the serum levels of RF, compared with the control group, whereas injection with CFA/MTX and CFA/TCA, significantly reduced the elevation of this marker in serum, compared with the CFA group. The efficacy of both treatments is similar to each other. TCA control had no effect on serum RF level (Fig. 2).

### TCA attenuated CFA-induced structural alteration of the hind limb

**Fig. 3:**
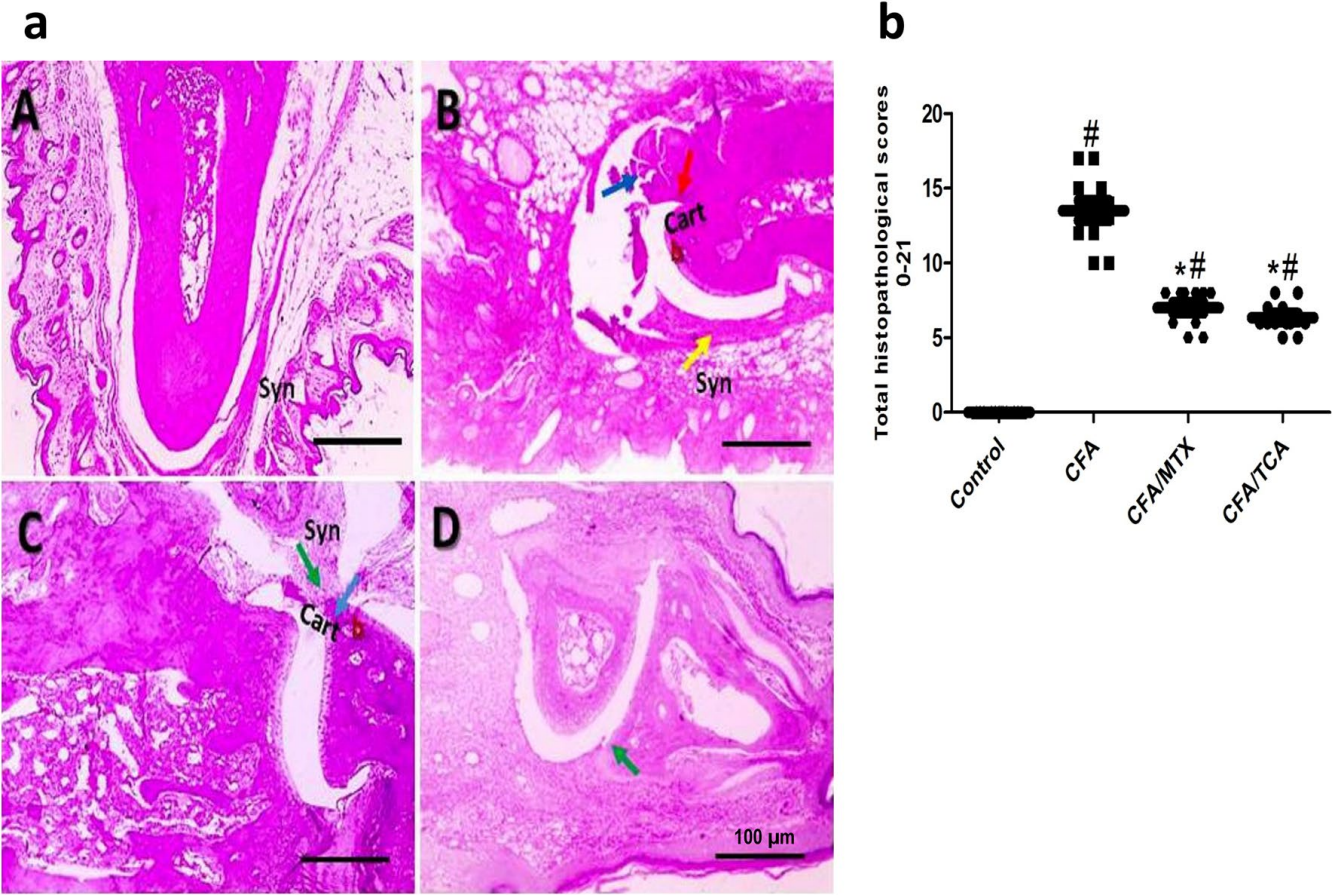
TCA alleviated CFA-induced structural alteration in hind paw joints after 5 weeks **a** Representative photomicrographs of H&E stained sections from hind paw joints showed normal synovial membrane (syn), articular cartilaginous surface (Cart) and underlying bone (**b**) in control group (**A**). Sections from hind paw joints in CFA group (**B**) showed marked synovial hyperplasia and inflammation (yellow arrow) with signs of cartilage and bone destruction (blue arrow) and marked proliferative synovial hyperplasia (red arrow) and increased vascularity in synovial membrane. Sections from hind paw joints in CFA/MTX group (**C**) showed mild proliferative synovial hyperplasia (green arrow), and vacuolations of chondrocytes (blue arrow). Sections from hind paw joints in CFA/TCA group (**D**) showed mild proliferative synovial hyperplasia in synovial membrane (green arrow). X:100 bar 100 µm. **b** Statistical analysis of the total histopathological scores from each H&E stained section/mouse. The data presented are the median (*n* = 6), #, *p < 0.05 compared with control, and CFA groups, respectively using Kruskal–Wallis test followed by Dunn's multiple comparison test, *CFA* complete freund’s adjuvant, *MTX* methotrexate, *TCA* trans-cinnamaldehyde

In control group, H&E stained sections from hind paw joints show normal synovial membrane, articular cartilaginous surface and underlying bone. The results show that chronic arthritis and related manifestations were obvious in the CFA group, which represent marked synovial hyperplasia and inflammation with signs of cartilage and bone destruction, and marked proliferative synovial hyperplasia and increased vascularity in synovial membrane. Compared to CFA group, these manifestations are decreased in CFA/MTX and CFA/TCA groups. Mild proliferative synovial hyperplasia, and vacuolations of chondrocytes are observed in CFA/MTX group and mild proliferative synovial hyperplasia is observed in CFA/TCA group (Fig. 3a). This was confirmed semiquantitavely through recording the total histopathological scores for all groups, which reveals that there is a significant reduction in the arthritis severity of synovial membrane and its underlying layers in all treated groups, compared with CFA group (Fig. 3b).

### The immunohistochemical changes of TCA on (NF-κB, TNF-α and COX-2)

**Fig. 4:**
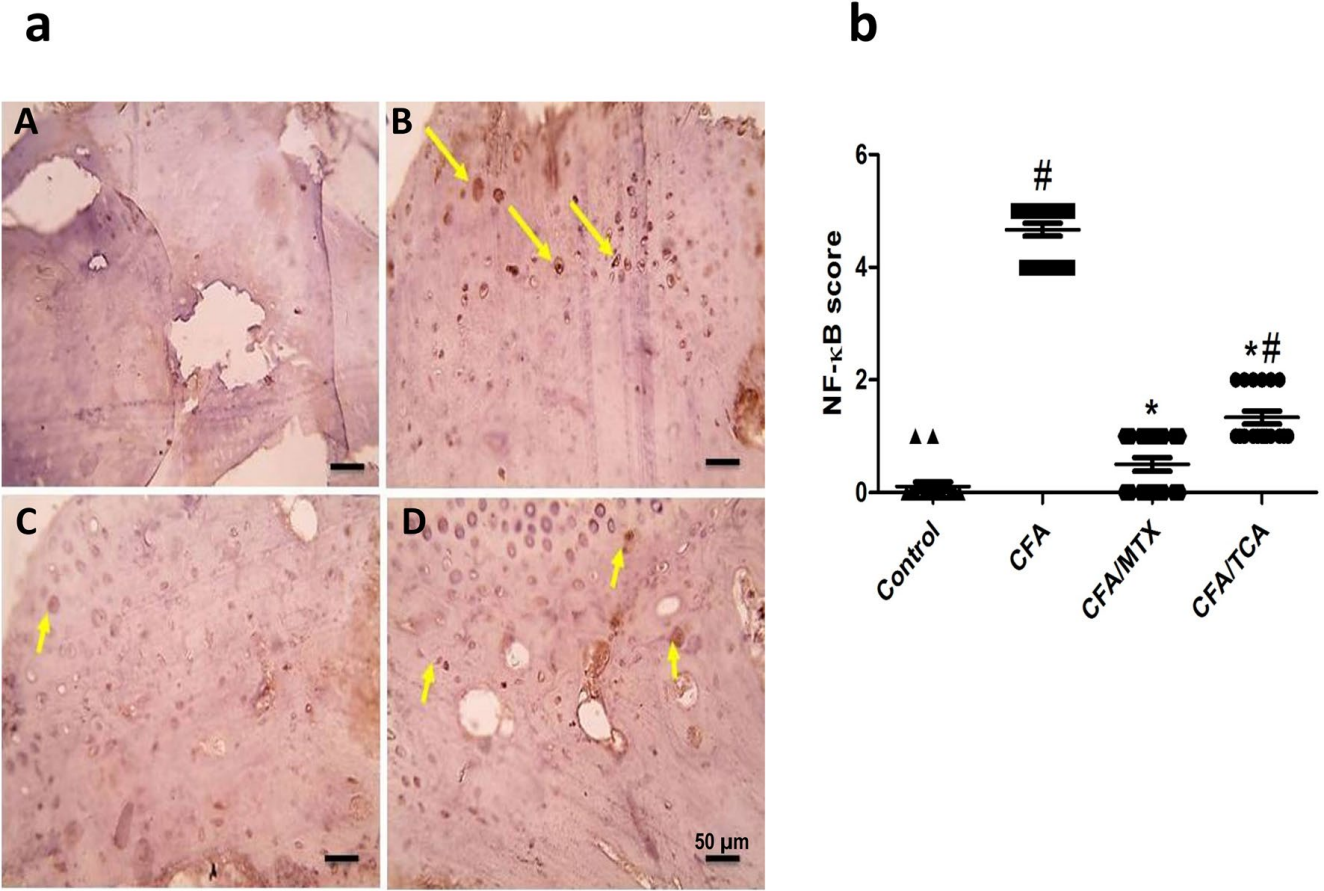
Effect of TCA on NF-kB expression in CFA-induced arthritis mice model **a** Representative photomicrographs of immunohistochemical analysis of NF-kB in paw sections from: control (**A**), CFA (**B**), CFA/MTX (**C**), and CFA/TCA (**D**) groups, after 5 weeks. IHC counterstained with Mayer's hematoxylin, X: 400, bar: 50 µm. **b** Scatter dot plot showing the score of NF-kB. The data presented are the median (*n* = 6), #, *p < 0.05 compared with control and CFA groups, respectively using Kruskal–Wallis test followed by Dunn's multiple comparison test, *CFA* complete freund’s adjuvant, *MTX* methotrexate, *TCA* trans-cinnamaldehyde, *NF-kB* nuclear factor-kappa beta

Compared with the control group, the expression of NF-kB in chondrocytes in articulating cartilage of CFA group is significantly enhanced (yellow arrows). In addition, compared with the CFA group, there is a significant marked reduction in the expression of NF-kB in mice CFA/MTX group, which was near normal values, in addition to a significant moderate reduction in NF-kB expression in CFA/TCA group (Fig. 4a, b). IHC counterstained with Mayer's hematoxylin, X:400 bar 50 µm.

**Fig. 5:**
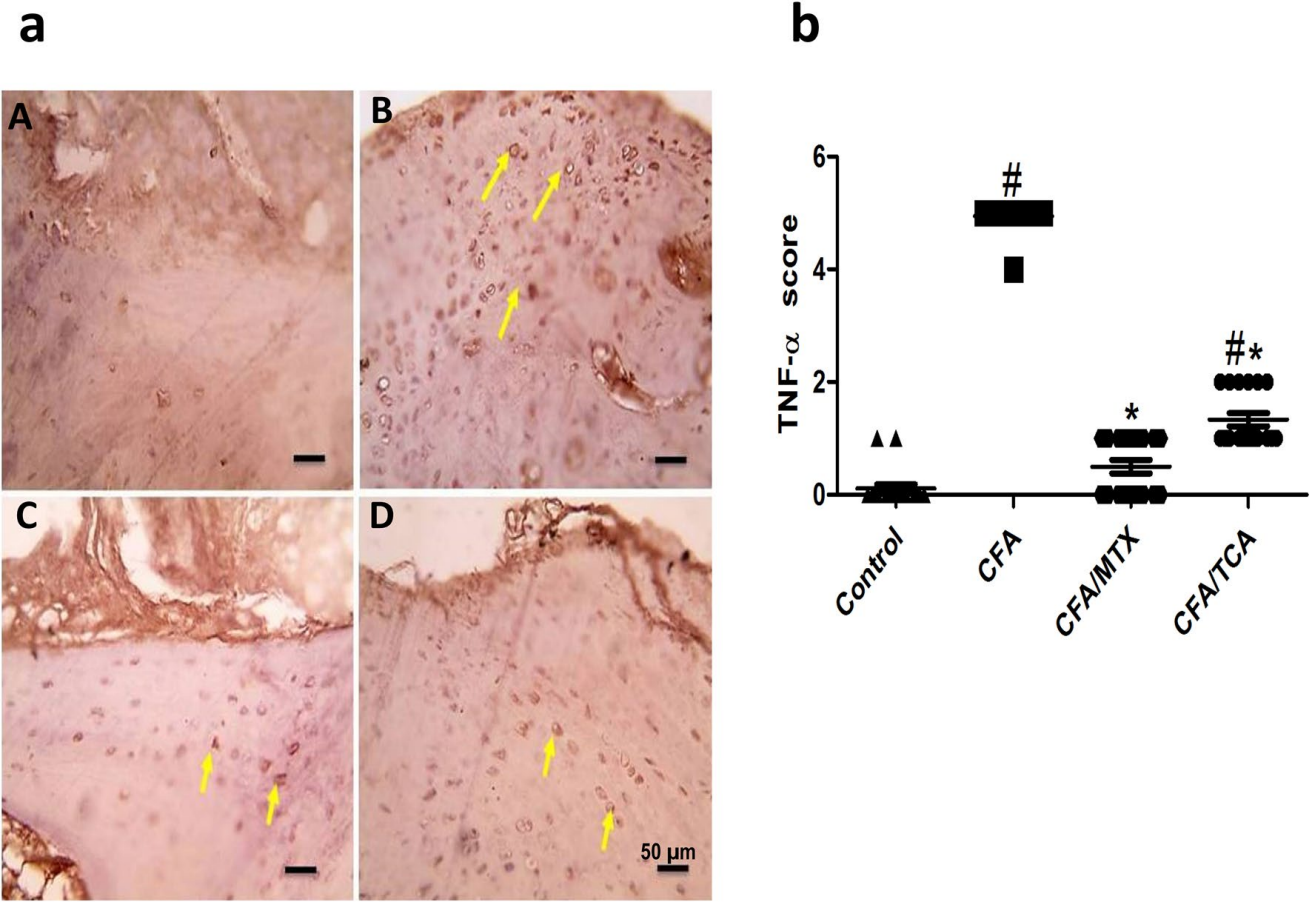
Effect of TCA on TNF-α expression in CFA-induced arthritis mice model **a** Representative photomicrographs of immunohistochemical analysis of TNF-α in paw sections from: control (**A**), CFA (**B**), CFA/MTX (**C**), and CFA/TCA (**D**) groups, after 5 weeks. IHC counterstained with Mayer's hematoxylin, X: 400, bar: 50 µm. **b** Scatter dot plot showing the score of TNF-α. The data presented are the median (*n* = 6), #, *p < 0.05 compared with control and CFA groups, respectively using Kruskal–Wallis test followed by Dunn's multiple comparison test, *CFA* complete freund’s adjuvant, *MTX* methotrexate, *TCA* trans-cinnamaldehyde, *TNF-α* tumor necrosis factor-alpha

Compared with the control group, the expression of TNF-α in chondrocytes in articulating cartilage of CFA group is significantly enhanced (yellow arrows). In addition, compared with the CFA group, there is a significant marked reduction in the expression of TNF-α in mice CFA/MTX group, which was near normal values, in addition to a significant moderate reduction in TNF-α expression in CFA/TCA group (Fig. 5a, b). IHC counterstained with Mayer's hematoxylin, X:400 bar 50 µm.

**Fig. 6:**
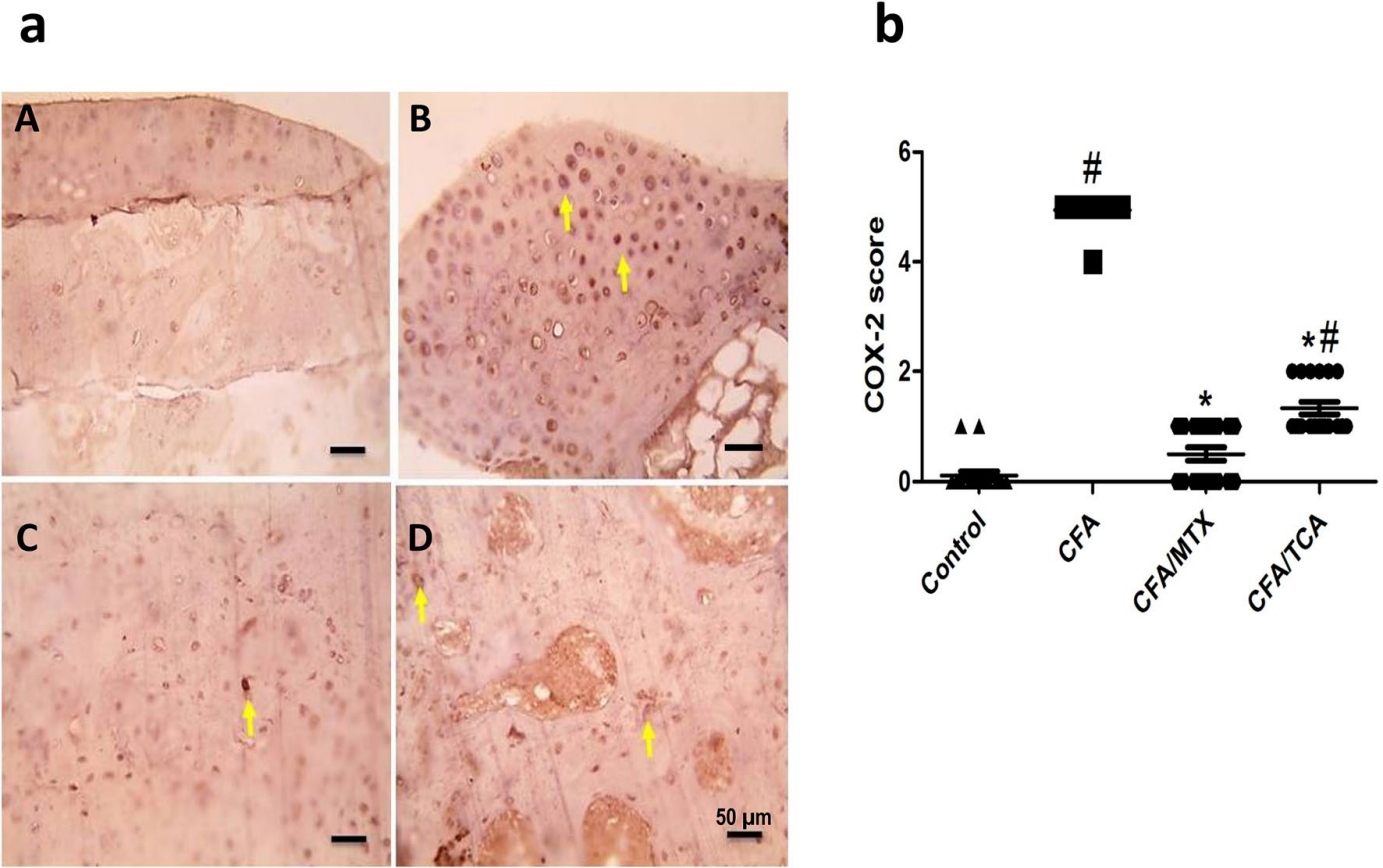
Effect of TCA on COX-2 expression in CFA-induced arthritis mice model **a** Representative photomicrographs of immunohistochemical analysis of COX-2 in paw sections from: control (**A**), CFA (**B**), CFA/MTX (**C**), and CFA/TCA (**D**) groups, after 5 weeks. IHC counterstained with Mayer's hematoxylin, X: 400, bar: 50 µm. **b** Scatter dot plot showing the score of COX-2. The data presented are the median (*n* = 6), #, *p < 0.05 compared with control and CFA groups, respectively using Kruskal–Wallis test followed by Dunn's multiple comparison test, *CFA* complete freund’s adjuvant, *MTX* methotrexate, *TCA* trans-cinnamaldehyde, *COX-2* cyclooxygenase-2

Compared with the control group, the expression of COX-2 in chondrocytes in articulating cartilage of CFA group is significantly enhanced (yellow arrows). In addition, compared with the CFA group, there is a significant marked reduction in the expression of COX-2 in mice CFA/MTX group, which was near normal values, in addition to a significant moderate reduction in COX-2 expression in CFA/TCA group (Fig. 6a, b). IHC counterstained with Mayer's hematoxylin, X:400 bar 50 µm.

### TCA alleviated CFA-induced arthritis dependent on the interleukin cascade (IL-1β/IL-6/IL-23/IL-17) through promotion of the immunosuppressive and anti-inflammatory activities

**Fig. 7:**
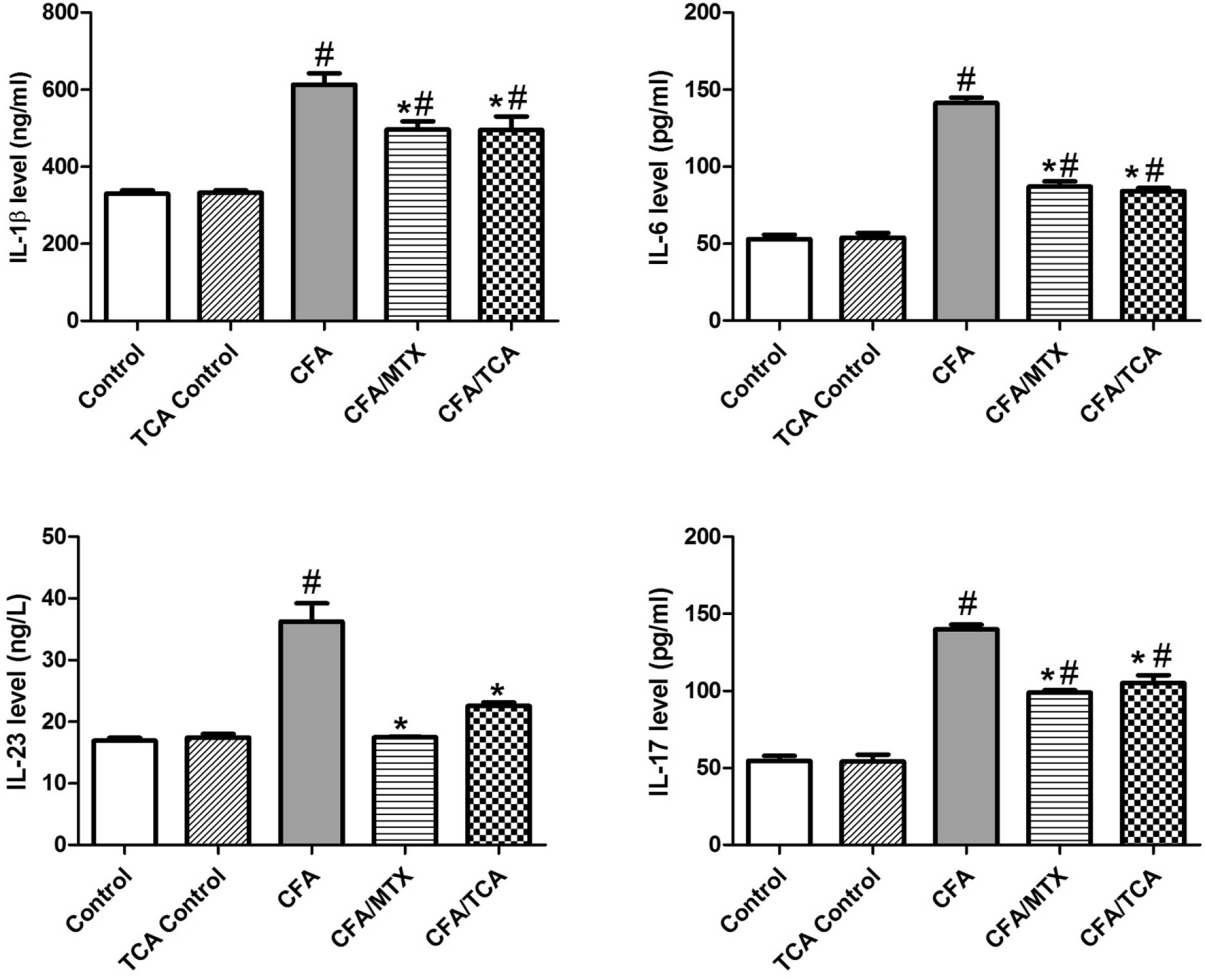
TCA alleviated CFA-induced arthritis dependent on interleukin cascade In CFA-induced arthritis mice model, after 5 weeks, levels of the inflammatory cytokines: IL-1β, IL-6, IL-23 and IL-17, were measured in paw tissues homogenates. Values are represented as mean ± SEM (*n* = 5). Data were statistically analyzed using one-way analysis of variance test (ANOVA) followed by Tukey's multiple comparison test. #, *p ≤ 0.05 vs. control and CFA groups, respectively, *CFA* complete freund’s adjuvant, *MTX* methotrexate, *TCA* trans-cinnamaldehyde, *IL* interleukin

Mice of CFA group show a significant increase in limb levels of IL-1β, IL-6, IL-23, and IL-17, compared to the control group. Contrarily, CFA/MTX and CFA/TCA treated groups, display a significant reduction in the levels of these inflammatory cytokines, compared with the CFA group, while both treated groups did not differ significantly from each other. TCA control had a near normal effect on the interleukin levels (Fig. 7).

## Discussion

In this research, CFA obviously produced RA, indicated by the appearance of all RA-related serological, morphological, and histological manifestations. An elevation in serum RF level was detected in CFA mice, which provides an excellent and simple tool to diagnose RA. Various studies reported similar results with this arthritis marker (Patil, Patil et al. 2011, Ananth et al. 2016, Xu et al. 2017, Gokhale et al. 2019, Zhu et al. 2020, Gad et al. 2021). TCA at a dose of 100 mg/kg, can decrease the progression of RA, indicated by the significant reduction in serum RF levels. A similar effect was observed with MTX at a dose of 0.75 mg/kg.

Herein, on a weekly basis, the effect of treatments on arthritis progression was estimated. Our results revealed that the CFA-induced paw swelling and inflammation were gradually ameliorated by TCA treatment to the same extent exhibited by MTX. This was indicated by the marked reduction in arthritis scores and paw thickness in both treatments from the 3rd week till the end of the study. In agreement with the marked reduction of paw swelling, the alleviation of arthritis progression by TCA was further examined by the histopathological analysis of paw tissues of mice. Both MTX and TCA, improved arthritic histopathological changes in CFA mice, including swelling, inflammatory infiltration, synovial hyperplasia, proliferation, cartilage, and bone erosion. Pathological scores of TCA and MTX mice had a lower degree of synovial hyperplasia compared to the scores of CFA mice. Therefore, the current results advocated the TCA potential to attenuate arthritis progression and reduce joint inflammation and destruction in CFA mice, as supported previously with former studies on cinnamaldehyde in collagen-induced arthritis (Cheng et al. 2020).

In light of this anti-arthritic effect of TCA, the research investigated the effect of TCA on various immune modulators and inflammatory mediators in CFA mice. The regulation of immune/inflammatory responses is considered one of the most vital explanatory mechanisms for the true event implicated in RA injury and was confirmed previously in various kinds of literature and experimental studies on adjuvant-induced arthritis models (Barsante et al. 2005, Xu et al. 2017, Kamel et al. 2018). Treatment with immunosuppressants in RA is well established (Shen et al. 2013a). Nuclear factor-kappa beta (NF-кB) is the main transcription factor involved in the regulation of immune/inflammatory responses. It upregulates receptors needed for immune cell function, and induction of the transcription of inflammatory cytokines, including TNF-α, IL-1β, IL-6, and other inflammatory mediators, such as COX-2 (Peng et al. 2012). The overproduction of NF-κB, which resulted in overexpression of these inflammatory mediators, all are greatly responsible for the development of arthritis, and were documented in multiple studies on human and animals (Tak and Firestein 2001; Yamamoto and Gaynor 2001; Hanada and Yoshimura 2002; Fishman et al. 2006; Shen et al. 2013a; Kamel et al. 2018). Additional studies confirmed that COX-2 expression promoted the inflammatory cytokine-mediated cartilage erosion, in CFA-and collagen-induced arthritis in rats (Peng et al. 2012; Alaaeldin et al. 2021). This mediated inflammatory and immune responses in RA were confirmed in our investigation by remarked nuclear translocation of NF-κB, TNF-α, and COX-2 in the affected joint of CFA mice. While, TCA and MTX-treated animals exerted a marked decline in their nuclear translocation, accompanied by remarkable reduction in the grading scores of NF-κB, TNF-α, and COX-2 in the inflamed joints. In support, previous research affirmed that MTX alleviated the expression of NF-κB, TNF-α, and COX-2 in CFA rats (Gowayed et al. 2020) and partly regulated serum COX‐2 activity in RA patients (Mello et al. 2000). TCA exhibits anti-inflammatory actions verified by various reports, which declared that TCA inhibited inflammation in *invivo* and *in-vitro* models by suppressing NF-κB in osteoarthritis rats (Xia et al. 2019). In addition, cinnamon metabolite attenuated the activation of NF-κB in experimental autoimmune encephalomyelitis (EAE) mice (Pahan and Pahan 2020). NF-κB suppression repressed the downstream inflammatory mediators as proved in an *in-vitro* experiment on osteoarthritis, which reported the TCA-induced decline in TNF-α and COX-2 levels (Wu et al. 2020).

In this context, some cytokines that can be released either by T cells, or by monocytes/macrophages, including TNF, IL-1, IL-6, and IL-17, interact to generate a cascade of cytokines, which induce chronic inflammatory responses and are involved in the etiology and progression of RA (Xu et al. 2017). Previous studies affirmed that cytokines produced from lymphocytes and macrophages in the synovial tissue exerted a complex and vital role in the pathogenesis and persistence of RA (Boissier, Assier et al. 2008; Burmester et al. 2014; Hu et al. 2014; Sokolove et al. 2014; Kumar et al. 2020). Additional reports confirmed that TNF-α, IL-1β and IL-6 are the main pathophysiological factors implicated in RA (Skapenko et al. 2005, Fishman et al. 2006; Han et al. 2013; Shen et al. 2013a, Xu et al. 2017). Moreover, former reports documented that serum and synovial levels of TNF-α and IL-17 were elevated in RA (Ziolkowska et al. 2000; Brennan and McInnes 2008). IL-6 and IL-23 activated the development of T helper-17 cells (Th-17), thereby, initiating the release of IL-17 by Th-17 cells (Chen and Zhou 2015). IL-23 and IL-17 were simultaneously upregulated in various injury models, including RA (Brennan and McInnes 2008; Duvallet et al. 2011). Previous work stated that IL-17 could activate NF-κB gene expression in CFA mice (Iwakura et al. 2011; Kamel et al. 2018), in addition to another clinical research that supported the therapeutic effect of anti-IL-17 therapy (Genovese et al. 2010). Under these facts, together with our histologic and immunohistochemical results, the present study declared that CFA-induced inflammation, indicated by the generation of immune reactions presented by elevation in the levels of IL-1β, IL-6, IL-23, and IL-17 in paw tissues. Contrarily, TCA and MTX can antagonize joint inflammation in CFA mice, as demonstrated by the reversal of all the elevated levels of these inflammatory cytokines in the inflamed paw tissue. Similar effects of MTX and TCA were reported previously. MTX could decrease plasma levels of TNF-α, IL-1β, IL-6, IL-17, and IL-23 in RA patients (Lina et al. 2011), in addition to serum levels of TNF-α, and IL-6 in CFA rats (Azouz et al. 2020). Meanwhile, cinnamaldehyde significantly suppressed the expressions of pro-inflammatory cytokines: interleukin IL-1β, IL-6, and TNF-*α* in the synovium of adjuvant arthritis rats (Liu et al. 2020), and in collagen-induced arthritis (CIA) rats (Cheng et al. 2020). In addition, cinnamaldehyde was reported to be a possible cure for immune/inflammatory diseases, and Th-17 cells can be the main player in its therapy. Cinnamaldehyde and cinnamon abrogated the expansion of Th-17 cells, thus reducing the gene expression of IL-17 in ulcerative colitis (Qu et al. 2021), and EAE (Pahan 2015; Pahan and Pahan 2020) mice models, respectively.

To our knowledge, there are no prior studies of the effect of TCA on IL-23 and IL-17 levels in the CFA-induced RA model in mice. Therefore, we presented the first *invivo* bioluminescence and fluorescence evidence that TCA alleviated the IL-23/IL-17 immune/inflammatory responses in the CFA-induced arthritis model. Consequently, treatment with TCA markedly enhanced the immune function in mice, monitored the production of pro-inflammatory cytokines, and improved the RA-associated inflammatory features, thereby, hampering the progression of arthritis.

## Conclusions

In summation, our data indicated that TCA as a dietary natural product had profound therapeutic effects on CFA-induced RA in mice, basically similar to those of the common anti-RA drug, MTX. TCA exerted its actions, partially through the improvement of the arthritic joint swelling, and histology, as it reduced synovial proliferation, diminished cartilage and bone erosion. In addition, it repressed immune/inflammatory signals of TNF-α, IL-1β, IL-6, IL-23, IL-17, and COX-2, which are in parallel with NF-κB modulation. Therefore, these effects outline the basis for further exploration of additive mechanisms implicated in the anti-arthritic actions of TCA in other models, as well**.**

## Data Availability

The datasets analyzed during the current study are available from the corresponding author on reasonable request.

## Abbreviations

CD4^+^T: Cluster of differentiation-4 T lymphocytes
CFA: Complete freund’s adjuvant
CMC: Carboxy methyl cellulose
COX-2: Cyclooxygenase-2
H&E: Hematoxylin and eosin
MTX: Methotrexate
NF-κB: Nuclear factor kappa beta
RA: Rheumatoid arthritis
TCA: Trans-cinnamaldehyde
TNF-α: Tumor necrosis factor-alpha
